# High levels of modified ceramides are a defining feature of murine and human cancer cachexia

**DOI:** 10.1002/jcsm.12626

**Published:** 2020-10-08

**Authors:** Pauline Morigny, Julia Zuber, Mark Haid, Doris Kaltenecker, Fabien Riols, Joanna D.C. Lima, Estefania Simoes, José Pinhata Otoch, Sören Fisker Schmidt, Stephan Herzig, Jerzy Adamski, Marilia Seelaender, Mauricio Berriel Diaz, Maria Rohm

**Affiliations:** ^1^ Institute for Diabetes and Cancer Helmholtz Center Munich Neuherberg Germany; ^2^ Joint Heidelberg‐IDC Translational Diabetes Program, Inner Medicine 1 Heidelberg University Hospital Heidelberg Germany; ^3^ German Center for Diabetes Research (DZD) Neuherberg Germany; ^4^ Molecular Metabolic Control Technical University of Munich Munich Germany; ^5^ Research Unit Molecular Endocrinology and Metabolism Helmholtz Center Munich, German Research Center for Environmental Health Neuherberg Germany; ^6^ Cancer Metabolism Research Group, LIM 26 HC, Medical School University of São Paulo São Paulo Brazil; ^7^ Experimentelle Genetik Technical University of Munich Freising‐Weihenstephan Neuherberg Germany; ^8^ Department of Biochemistry, Yong Loo Lin School of Medicine National University of Singapore Singapore

**Keywords:** Cancer cachexia, Lipidomics, Signalling lipids, Sphingolipids, Ceramides

## Abstract

**Background:**

Cancer cachexia (CCx) is a multifactorial energy‐wasting syndrome reducing the efficiency of anti‐cancer therapies, quality of life, and survival of cancer patients. In the past years, most studies focused on the identification of tumour and host‐derived proteins contributing to CCx. However, there is still a lack of studies addressing the changes in bioactive lipids. The aim of this study was to identify specific lipid species as a hallmark of CCx by performing a broad range lipid analysis of plasma from well‐established CCx mouse models as well as cachectic and weight stable cancer patients.

**Methods:**

Plasma from non‐cachectic (PBS‐injected mice, NC26 tumour‐bearing mice), pre‐cachectic and cachectic mice (C26 and LLC tumour‐bearing mice, Apc^Min/+^ mutant mice), and plasma from weight stable and cachectic patients with gastrointestinal cancer, were analysed using the Lipidyzer™ platform. In total, 13 lipid classes and more than 1100 lipid species, including sphingolipids, neutral and polar glycerolipids, were covered by the analysis. Correlation analysis between specific lipid species and readouts of CCx were performed. Lipidomics data were confirmed by gene expression analysis of metabolic organs to analyse enzymes involved in sphingolipid synthesis and degradation.

**Results:**

A decrease in several lysophosphatidylcholine (LPC) species and an increase in numerous sphingolipids including sphingomyelins (SMs), ceramides (CERs), hexosyl‐ceramides (HCERs) and lactosyl‐ceramides (LCERs), were mutual features of CCx in both mice and cancer patients. Notably, sphingolipid levels gradually increased during cachexia development. Key enzymes involved in ceramide synthesis were elevated in liver but not in adipose, muscle, or tumour tissues, suggesting that ceramide turnover in the liver is a major contributor to elevated sphingolipid levels in CCx. LPC(16:1), LPC(20:3), SM(16:0), SM(24:1), CER(16:0), CER(24:1), HCER(16:0), and HCER(24:1) were the most consistently affected lipid species between mice and humans and correlated negatively (LPCs) or positively (SMs, CERs and HCERs) with the severity of body weight loss.

**Conclusions:**

High levels of sphingolipids, specifically ceramides and modified ceramides, are a defining feature of murine and human CCx and may contribute to tissue wasting and skeletal muscle atrophy through the inhibition of anabolic signals. The progressive increase in sphingolipids during cachexia development supports their potential as early biomarkers for CCx.

## Introduction

Cancer cachexia (CCx) is a multifactorial energy‐wasting syndrome characterized by an unintentional loss of body weight, reflecting skeletal muscle and adipose tissue wasting.^1^ CCx implies a poor prognosis as it affects the tolerance and efficiency of anti‐cancer therapies and thereby reduces the quality of life and survival of cancer patients. CCx is present in 50–80% of cancer patients and is estimated to be directly responsible for at least 20% of cancer‐related deaths.^2^ Classical features of CCx include anorexia, chronic inflammation, acute phase response in the liver, resistance to anabolic signals, and general catabolic state.^3^ However, body weight loss in CCx cannot be fully reversed by nutritional approaches, and despite efforts to find efficient therapeutic options,^4^ no defined standard of care is currently available to counteract tissue wasting. In order to find novel therapies, previous studies have focused on the identification of tumour and host‐derived proteins involved in tissue wasting, and thereby identified several cachexia mediators including pro‐inflammatory cytokines such as IL6, TNFα, or IL1α/β; members of the TGFβ superfamily; or zinc‐α2‐glycoproteins.^5^ However, none of them has yet led to an efficient routine therapy.

Lipids are another type of bioactive molecules involved in inter‐organ communications. As essential components of the plasma membrane, lipids adopt a central role in the transmission of extracellular signals. For example, upon cancer incidence, an increased amount of cholesterol‐enriched and sphingolipid‐enriched signalling platforms (i.e. lipid rafts) in the plasma membrane promotes cancer cell survival, migration, and invasion.^6^ Furthermore, plasma membrane fluidity, which depends on the ratio of unsaturated/saturated fatty acids (FAs) in phospholipids composing the lipid bilayer, is essential for lipid raft dynamics and transmission of insulin signalling.^7^ Intracellular lipid species, such as ceramides (CERs) or diacylglycerols (DAGs), directly interfere with insulin signalling.^8^ In addition to their intracellular effects, lipids are also important endocrine mediators. Numerous circulating bioactive lipids have been identified in the past decades that control a plethora of essential biological functions also affected in CCx. These include inflammation and immune response (eicosanoids and glucocorticoids),^9^ glucose and lipid metabolism [glucocorticoids, fatty acid‐hydroxy‐fatty acids, and phosphatidylcholine (18:0/18:1)],^10^, ^11^, ^12^ as well as energy expenditure (12,13‐diHOME).^13^ Dysregulation of bioactive lipids is associated with many diseases including insulin resistance, type 2 diabetes, sepsis, cancer, atherosclerosis, and cardiovascular diseases. Therefore, modifications of bioactive lipid levels could affect inter‐organ communication and participate to the wasting syndrome in CCx. However, despite its importance, there is still a lack of available studies focusing on lipid alterations in CCx.

So far, a reduction in circulating lysophosphatidylcholine (LPC) levels,^14^ as well as alterations in free FA (FFA) quality,^15^ have been reported in cachectic cancer patients compared with weight stable cancer patients and were associated with inflammatory markers as well as the degree of body weight loss.^15^, ^16^ Nevertheless, to our knowledge, no detailed lipidomic analysis comparing plasma of cachectic cancer patients and different tumour‐bearing mouse models has been conducted so far. The aim of this study was to deepen our knowledge of lipid metabolism in CCx by performing a broad range targeted lipid analysis on plasma from well‐established mouse models of CCx as well as cachectic and weight stable cancer patients via flow injection analysis (FIA)‐mass spectrometry/mass spectrometry (MS/MS) using the Lipidyzer™ platform. Our objective was to identify common, specific lipid species as a hallmark of cachexia, induced by various types of tumours in both mice and humans, within a translational approach. This study may pave the way for future research on lipids as biomarkers and mediators of CCx.

## Methods

### Animals

*In vivo* experiments were carried out using 8‐ to 12‐week‐old male BALB/c or C57BL/6J mice that were obtained from Charles River Laboratories (CRL, Brussels, Belgium). C57BL/6J‐APC^Min/+^ mice, as originally described by Moser *et al*.^17^ and later established as model of cachexia^18^ were purchased from the Jackson Laboratory (JAX stock #002020) and bred on a C57BL/6J background. All mice were maintained under specific pathogen‐free conditions on a 12‐h light–dark cycle at 22°C with unrestricted access to regular rodent chow diet (Kliba Nafag #3437, Provimi Kliba AG, Kaiseraugst, CH) and water. In each animal experiment, mice were assigned to groups in a manner that body weight, lean mass, and fat mass were similar between the groups as confirmed by non‐significant statistical analysis. Animal handling and experimentation were performed in accordance with the institutional animal welfare officer, and the necessary licences were obtained from the state ethics committee and government of Upper Bavaria (nos. 55.2‐2532.Vet_02‐16‐136 and ROB‐55.2‐2532.Vet_02‐18‐93). The number of mice used for each experiment is indicated in the figure legends.

In different experiments, mice were injected subcutaneously into the right flank with 2 million of Lewis lung cancer (LLC), 1 million of NC26, or 1 million of C26 cells, resuspended in Dulbecco's phosphate‐buffered saline (Thermo Fisher, #14190250). Non‐tumour healthy control mice were injected with 50 μL of Dulbecco's phosphate‐buffered saline. Mice were monitored for 2–3 weeks after tumour cell implantation, and tumour growth, body weight, and food intake were recorded daily. Mice were fasted for 6 h before killing. Mice were sacrificed by an overdose of ketamine/xylazine after having lost 10–15% of their initial body weight or reached humane endpoints (tumour >15 mm or ulceration of tumour). In the C26‐pre‐cachexia vs. C26‐cachexia study, the cachectic C26 tumour‐bearing group was defined by a 10–15% body weight loss, whereas the C26‐pre‐cachectic group was characterized by a similar tumour size but a non‐significant loss of body weight at the moment of sacrifice. Thus, mice from the two groups had a similar tumour size but a different degree of body weight loss. A C26‐pre‐cachectic control with a matching tumour size was euthanised on the same day as a cachectic C26 tumour‐bearing mouse.

Blood was withdrawn from the vena cava, transferred into EDTA tubes (Kabe Labortechnik, #78001) and centrifuged at 2000 *x* *g*, 4°C for 10 min. Plasma was divided into 50 μL aliquots, immediately snap frozen in liquid nitrogen and stored at −80°C until further processing. Tumours and organs including liver, inguinal white adipose tissue (WAT), epididymal WAT, heart, and gastrocnemius (GC) muscles were collected, snap frozen, and stored at −80°C.

Total cholesterol, HDL, LDL, glucose, and triacylglycerol (TAG) plasma levels were measured using a Beckman Coulter AU480 Chemistry Analyser.

### Patient cohorts and samples

Enrolment of Brazilian gastrointestinal cancer patients (stages I–IV) occurred at the Surgical Clinic of the University Hospital of São Paulo after signature of the fully informed consent. This study was approved by the Ethics Committee on Research Involving Human Subjects of the University of the São Paulo Biomedical Sciences Institute (CEP 1151/13 CAAE n 5493116.6.0000467) and by the University Hospital (CEP 1390/14 CAAE n 54930116.6.3001.0076) and is in accordance with the Declaration of Helsinki. Exclusion criteria: BMI > 29.9 kg/m^2^, chronic anti‐inflammatory therapy or chronic inflammatory processes not related to cachexia, chemotherapy treatment (at the time or recent past 5 years), AIDS, or liver or kidney failure. Approximately 20 mL of blood were collected in pre‐surgical fast at the hospital. Biochemical analysis was performed with the automatic LABMAX 240® equipment (Labtest, Lagoa Santa, Brazil) using commercial kits. Haemoglobin concentration was obtained from the hospital records, before the surgery. Cancer patients were classified as weight stable (Ws) or cachectic (Cx), following Evans *et al*.^19^

### Lipid extraction procedure

Lipid extraction was based on the protocol by Matyash *et al*.^20^ After thawing at room temperature (RT) for 30 min, 25 μL of plasma samples were transferred into 1.5 mL glass vials together with 75 μL of MilliQ water. For accurate quantification, 25 μL of a mix of 54 deuterated internal standards were then added to the samples (SCIEX, cat#: 5040156, lot#: LPISTDKIT‐101, LPISTDKIT‐102, LPISTDKIT‐102b). The certificate of analysis documents containing the exact composition of the mixes can be downloaded at the following links:

https://sciex.com/Documents/Downloads/Certificates%20of%20Analysis/lipidyzer/IS_Kit_5040156_LPISTDKIT_101.pdf

https://sciex.com/Documents/Downloads/Certificates%20of%20Analysis/lipidyzer/2018/IS_Kit_5040156_LPISTDKIT_102.pdf

https://sciex.com/Documents/Downloads/Certificates%20of%20Analysis/lipidyzer/2018/IS_Kit_5040156_LPISTDKIT_102b.pdf

For lipid extraction, 160 μL of methanol (MeOH, Optigrade, Thermo Fisher) and 575 μL methyl *tert*‐buthyl ether (MTBE) were added followed by incubation on an orbital shaker DOS‐10L (NeoLabLine, Heidelberg, Germany) at 250 rpm for 30 min. For phase separation, 200 μL of H_2_O was added to each vial. The mixtures were vortexed, and the vials were centrifuged at 5000 *x* *g* at RT using a Sigma 4‐5C centrifuge (Qiagen, Hilden, Germany) for 10 min. The upper (organic) phase was transferred into new glass vials and evaporated with nitrogen gas using a Barkey evaporator (Barkey, Leopoldshoehe, Germany).

The aqueous phase was again extracted with 100 μL MeOH and 300 μL MTBE. After an addition of 100 μL H_2_O, the samples were incubated at RT at 250 rpm for 10 min and then centrifuged at 5000 *x* *g* for 10 min. The organic phase was transferred into the respective vial from the first extraction step and evaporated to dryness with gaseous nitrogen.

Samples were reconstituted in 250 μL running solvent [10 mM ammonium acetate in dichloromethane:MeOH (50:50, v/v)], transferred into new vials with insert, and centrifuged at 4000 *x* *g* for 10 min.

Each substudy was prepared and measured in a separate batch. We generated a pooled QC sample containing small aliquots of each sample from the respective substudy. Within each batch, three aliquots of the QC pool samples and three blank samples (H_2_O) were extracted with the above described method. For further QC purposes, 25 μL human control plasma (SCIEX, cat#4368703) was spiked with 12.5 μL QC spike standards (SCIEX, cat#5040408) and extracted with the described method.

### Lipid measurements

All samples were measured with a Shimadzu Nexera X2 UHPLC‐system coupled to a SCIEX QTRAP 5500 mass spectrometer equipped with a SelexION differential ion mobility interface (SCIEX, Darmstadt, Germany) operated with Analyst 1.6.3. 50 μL of the re‐dissolved sample were injected using the running solvent at an isocratic flow rate of 7 μL/min. After 6 min, the flow rate was ramped to 30 μL/min for 2 min to allow for washing. Each sample was analysed using lipid specific multiple reaction monitoring transitions in two consecutive FIA runs. In the first run, phosphatidylcholines (PCs), LPCs, phosphatidylethanolamines (PEs), lysophosphatidylethanolamines (LPEs), and sphingomyelins (SMs) were separated with the SelexION differential mobility spectrometry (DMS) cell using field asymmetric ion mobility MS^21^ prior to ionization in the Turbo V source of the mass spectrometer. To enhance the separation of the lipids, 1‐propanol was used as chemical modifier. The DMS cell was operated with the following conditions: DMS temperature low, modifier composition low, separation voltage 3500 V, DMS resolution enhancement low.

In the second run, FFAs, TAGs, DAGs, CERs, dihydro‐ceramides, lactosyl‐ceramides (LCERs), hexosyl‐ceramides (HCERs), and cholesteryl esters (CEs) were measured with the DMS‐cell switched off. Dihydro‐ceramides were later excluded from the analysis as levels were below the detection threshold for most mouse samples.

The mass spectrometer was operated with the following conditions: curtain gas 17, heater gas 25, collisionally activated dissociation gas medium, ion spray voltage +4100 V in electrospray ionisation+ mode and − 4100 V in electrospray ionisation− mode, temperature 200°C.

Lipids were quantified with the Lipidyzer™ Workflow Manager (version 1.0) by calculating the area ratio between the analytes and the respective internal standards and subsequent multiplication with the known concentration of the IS. Per default, the Lipidyzer™ Workflow Manager presents the concentration in the dimension nmol/g. The concentration was then converted to nmol/mL assuming that 1 g of plasma is equal to 1 mL.^22^

Prior to each batch, the DMS cell was tuned with the SCIEX tuning mix (SCIEX, Darmstadt, Germany, cat # 5040141), and the stability of the instrument was checked with a system suitability test mix (SCIEX, Darmstadt, Germany, cat # 5040407). Each substudy was measured in a separate batch.

### Lipidomics data pre‐processing

Lipidomics data were pre‐processed using R (version 3.6.1).^23^ To assure high data quality, a multi‐step procedure was applied for each substudy:

First, a principal component analysis was used to detect and remove outlier samples prior to further data processing. In a second step, lipids that contained more than 50% of missing values over all samples were discarded. Next, lipids that had a coefficient of variation >25%, determined by the QC pool samples, were removed from the data set. The last quality control step comprised the calculation of the dispersion ratio (D‐ratio) for each lipid^24^:

$D.\text{ratio}=\frac{\sigma_{\text{tech}}}{\left(\sqrt{\sigma_{\text{biol}}^{2}+\sigma_{\text{tech}}^{2}\;}\;\right)}$ where $\sigma_{\text{tech}}^{2}$ is the technical variance determined by the variance of the QC pool samples and $\sigma_{\text{biol}}^{2}$ is the biological variance given by the variance of the biological samples within each substudy. We used a D‐ratio threshold of 50%, as a D‐ratio >50% implies that the technical variance is higher than the biological variance.

Missing values were imputed using the *k*‐nearest‐neighbour obs‐sel approach described by Do *et al*. with *k* = 3 nearest‐neighbours.^25^

### Quantitative PCR

Total RNA was extracted from frozen organs by performing a tissue lysis in TRIzol (Life Technologies, #15596018) with Qiagen's TissueLyser II (Qiagen, # 85300). RNA was then isolated by adding chloroform, precipitated with isopropanol, and washed two times with 75% ethanol before resuspension in ultrapure water. RNA concentration was quantified using a NanoDrop2000 spectrophotometer (Thermo Fisher, # ND‐2000) and total RNA (1 μg) was treated with DNase and reverse‐transcribed into cDNA (Qiagen, #205313) according to the manufacturer's recommendations. Real‐time–quantitative PCR was conducted using Applied Biosystems' QuantStudio 7 Flex Real‐Time PCR System (Applied Biosystems, # 4485695) and TaqMan Gene Expression Master Mix (Life Technologies, #4369514). RNA expression data were quantified according to the ΔCt method, as described previously,^26^ and normalized to levels of TATA‐box binding protein RNA (*Tbp*, Mm01277042_m1). The following Taqman probes (Applied Biosystems) targeting genes involved in CER synthesis and degradation were used: *Cers2* [Mm00504086_m1], *Cers5* [Mm00510998_m1], *Cers6* [Mm00556165_m1], *Degs1* [Mm00492146_m1], *Degs2* [Mm00510313_m1], *Sptlc1* [Mm00447343_m1], *Sptlc2* [Mm00448871_m1], *Smpd1* [Mm00448871_m1], *Kdsr* [Mm01290268_m1], *Asah1* [Mm00480021_m1], *Ugcg* [Mm00495925_m1], *St3gal5* [Mm00488237_m1].

### Western blot analysis

Proteins were extracted from frozen livers following lysis in ice‐cold lysis buffer (50 mM Tris/HCl pH 7.2, 1 mM EDTA, 1 mM DTT, 0.15 M NaCl, 1% NP‐40, 10 mM NaF, 2 mM Na_3_VO_4_, 1 X cOmplete protease inhibitor cocktail and 1 X PhosSTOP cocktail from Roche). 30 μg of protein extracts were separated on 4–20% *tris*‐glycine gels, blotted onto nitrocellulose membranes and incubated with the following primary antibodies: Cers6 (1/1000, Abnova #H00253782‐A01), Smpd1 (1/1000, Invitrogen #PA595730), and vinculin (1/10000, Abcam #ab129002). Anti‐rabbit or anti‐mouse IgG coupled to horseradish peroxidase were used as secondary antibodies, and immunoreactive proteins were determined by chemiluminescence using a ChemiDoc MP System (Bio‐Rad).

### Statistical analysis

Statistical analysis was performed using GraphPad Prism 8.3. Normality was tested using D'Agostino–Pearson and Shapiro–Wilk normality tests. Statistical tests were two sided. Unpaired Student's *t* tests and Mann–Whitney tests were performed to compare two conditions, whereas unpaired one‐way ANOVA or Kruskal–Wallis tests were performed to determine differences between several groups. Bonferroni's or Dunn's *post‐hoc* tests were applied when significant differences were found. Linear regression was used to test association between two variables. Wilcoxon rank sum tests with Benjamini–Hochberg correction for multiple comparison were conducted to statistically analyse fold changes in the heat maps. Comparable tumour staging, tumour location, and gender between weight‐stable and cachectic cancer patients were ensured using Fisher's exact test. Partial least squares‐discriminant analyses (PLS‐DAs) were conducted with MetaboAnalyst 4.0.^27^

## Results

### Plasma lipid profiles are altered in various mouse models of cancer cachexia

In the current study, we compared different commonly used mouse models of CCx characterized by various tumour entities, tumour sizes, and degrees of body weight loss. Circulating lipids were analysed by FIA‐MS/MS using the Lipidyzer™ platform. In total, 13 lipid classes including more than 1100 lipid species were covered by the analysis.

**Figure 1:**
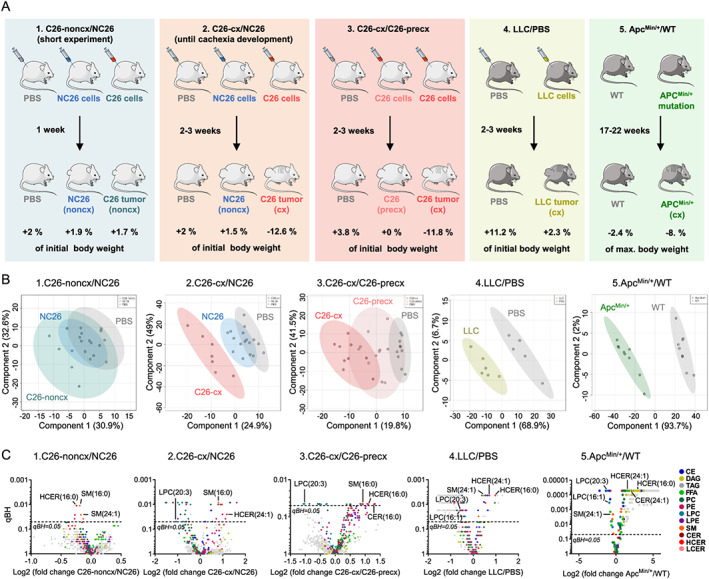
Lipidomic analysis of different mouse models of cachexia. (*A*) Diagram presenting the different mouse experiments conducted. *Experiment 1*—Comparison of healthy phosphate‐buffered saline (PBS)‐injected controls (grey, *n* = 6 animals), non‐cachectic NC26 (blue, *n* = 7 animals) and C26 (C26‐noncx, dark blue, *n* = 7 animals) tumour‐bearing mice 1 week after tumour implantation (small tumours). *Experiment 2*—Comparison of healthy PBS controls (grey, *n* = 8 animals), non‐cachectic NC26 (blue, *n* = 9 animals), and cachectic C26 (C26‐cx, red, *n* = 7 animals) tumour‐bearing mice 2–3 weeks after tumour implantation. *Experiment 3*—Comparison of PBS controls (grey, *n* = 10 animals), pre‐cachectic (C26‐precx, pink, big tumours but no body weight loss yet, *n* = 11 animals), and cachectic (C26‐cx, red, big tumours and body weight loss, *n* = 9 animals) C26 tumour‐bearing mice 2–3 weeks after tumour implantation. *Experiment 4*—Comparison of healthy PBS controls (for the legend of experiment 3) (grey, *n* = 5 animals) and cachectic LLC tumour‐bearing mice (yellow, *n* = 6 animals) 2–3 weeks after tumour implantation. *Experiment 5*—Comparison of healthy wildtype (WT, grey, *n* = 9 animals) controls and cachectic Apc^Min/+^ mutant mice (green, *n* = 9 animals). (*B*) Partial least squares‐discriminant analysis (PLS‐DA) score plots for plasma lipid species showing discrimination between the different groups of each experiment. PLS‐DAs were calculated using MetaboAnalyst 4.0. Ellipses represent 95% confidence intervals for each individual group. (*C*) Volcano plots showing changes in plasma lipid species between C26‐noncx/NC26 tumour‐bearing mice, C26‐cx/NC26 tumour‐bearing mice, C26‐cx/C26‐precx tumour‐bearing mice, LLC tumour‐bearing mice/PBS controls, and mutant Apc^Min/+^ mice/WT mice. The dashed lines indicate adjusted *P* value (qBH) of 0.05. Statistical analyses were performed using two‐sided Wilcoxon rank sum tests. *P* values were adjusted for multiple testing using the Benjamini–Hochberg correction method.

In a first control experiment, mice were either injected subcutaneously with the cachexia‐inducing colon cancer cell line C26 or the non‐cachexia‐inducing control colon cancer cell line NC26. Mice were sacrificed 1 week after tumour cell injection, while tumours were still small and no cachectic phenotype was observed, i.e. no loss of body weight, adipose tissue, skeletal or cardiac muscles (*Figures* 1A and S1A–S1F). As expected at this early stage of the disease, the lipid profile of non‐cachectic C26 tumour‐bearing mice (C26‐noncx, dark blue colour code) did not discriminate from the lipid profiles of non‐cachectic NC26 tumour‐bearing (blue colour code) and phosphate‐buffered saline (PBS)‐injected mice (grey colour code) by PLS‐DA (*Figure* 1B, experiment 1). In two additional experiments, we compared cachectic C26 tumour‐bearing mice (C26‐cx, red colour code) either to non‐cachectic NC26 (experiment 2) or to pre‐cachectic (i.e. before the onset of weight loss) C26 tumour‐bearing mice (C26‐precx, pink colour code, experiment 3) (*Figure* 1A). In contrast to the first experiment, all mice from the latter two experiments carried bigger tumours of similar sizes (*Figure* S1B), but only C26‐cx mice had significant reduction in body weight, adipose tissue, GC muscles, and heart mass (*Figures* S1A and S1C–S1F). Interestingly, a PLS‐DA separated the circulating lipid profile of C26‐cx mice from the lipid profile of non‐cachectic NC26 mice, which was overlapping with the control PBS‐injected mice (*Figure* 1B, experiment 2). C26‐precx mice had an intermediate lipid profile between non‐cachectic PBS and cachectic C26‐cx mice that reflected the intermediate body weight loss observed (*Figure* 1B, experiment 3). To strengthen our observations, we analysed two other well‐established mouse models of CCx (*Figure* 1A, experiments 4 and 5). Both, subcutaneous injection of Lewis lung carcinoma cells (LLC, yellow colour code) and genetic mutation of the Apc locus (Apc^Min/+^, green colour code), the latter inducing spontaneous intestinal tumourigenesis with ageing,^17^ led to a strong modification in the circulating lipid profile associated with the development of cachexia as indicated by PLS‐DA (*Figures* 1B and S1A–S1F, experiments 4 and 5). Volcano plots highlighted deep modifications in the quality and quantity of circulating lipid species in cachectic mice compared with non‐cachectic healthy PBS and NC26 control mice as well as C26‐precx mice (*Figure* 1C). Importantly, we found mutual features of cachexia among our different mouse models, such as an increase in several sphingolipids (i.e. SMs, CERs, and HCERs) or a reduction in LPCs. Strikingly, in the C26‐noncx mice, the respective lipid classes were unaffected or even regulated in an opposite manner, underlining that these lipids are specifically dysregulated in the course of cachexia.

### High circulating amounts of sphingolipids is a common feature of cancer cachexia in mice

**Figure 2:**
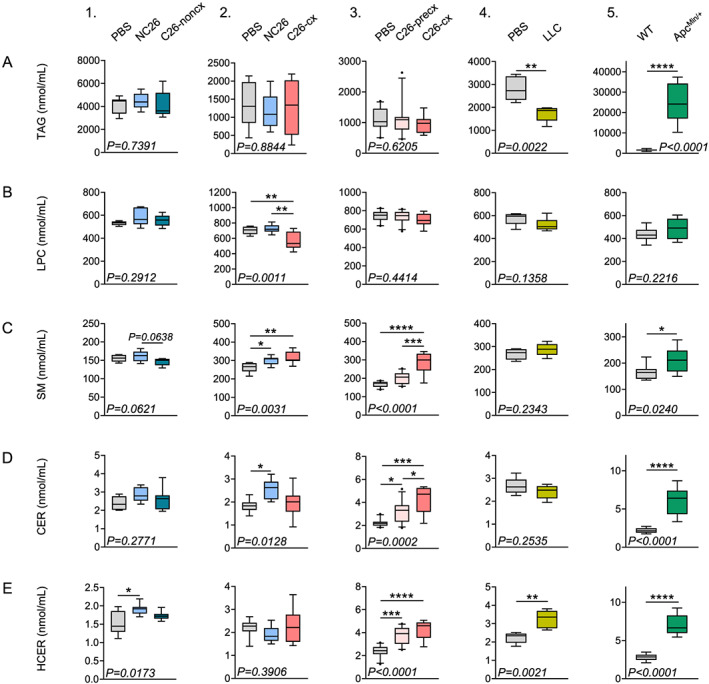
Various lipid classes are altered in plasma of cachectic mice. (*A*–*E*) Plasma lipid class sum concentrations for each experiment. Triacylglycerols (TAG) (*A*), lysophosphatidylcholine (LPC) (*B*), sphingomyelins (SM) (*C*), ceramides (CER) (*D*), hexosyl‐ceramide (HCER) (*E*). From left to right: *Experiment 1*—Phosphate‐buffered saline (PBS) (grey boxplots, *n* = 6 animals), non‐cachectic NC26 (blue boxplots, *n* = 7 animals), and C26 (C26‐noncx, dark blue boxplots, *n* = 7 animals) tumour‐bearing mice. *Experiment 2*—PBS (grey boxplots, *n* = 8 animals), non‐cachectic NC26 (blue boxplots, *n* = 9 animals), and cachectic C26 tumour‐bearing mice (C26‐cx, red boxplots *n* = 7 animals). *Experiment 3*—PBS (grey boxplots, *n* = 10 animals), pre‐cachectic (C26‐precx, pink boxplots, *n* = 11 animals) and cachectic (C26‐cx, red boxplots, *n* = 9 animals) C26 tumour‐bearing mice. *Experiment 4*—PBS (grey boxplots, *n* = 5 animals) and cachectic LLC tumour‐bearing mice (yellow boxplots, *n* = 6 animals). *Experiment 5*—Wildtype (WT, grey boxplots, *n* = 9 animals) and cachectic Apc^Min/+^ mutant mice (green boxplots, *n* = 9 animals). Data are median 10–90 percentile. Statistical analyses were performed using unpaired one‐way ANOVA or Kruskal–Wallis tests with Bonferroni or Dunn's *post‐hoc* tests respectively (experiments 1–3) and unpaired *t* test or Mann and Whitney test (experiments 4–5). Tests were two sided. *P < 0.05, **P < 0.01, ***P < 0.001, ****P < 0.0001.

We then focused on changes in the main lipid classes during cachexia development. Plasma neutral lipids exhibited contrasting regulation between the different models (*Figures* 2A and S2A–S2C). While cholesteryl esters (CEs) and DAGs were unchanged in the C26‐cx and LLC models, both classes were very significantly increased in Apc^Min/+^ mice. Total TAG levels were unchanged in C26‐cx mice, decreased in LLC mice, and extremely elevated in Apc^Min/+^ mice, confirming volcano plots shown previously in *Figure* 1C. These results were also validated using a serum analyser (*Table* S1). Total measured amounts of FFA and polar lipids, including PCs, PEs, LPCs and LPEs, were not consistently modified between the different mouse models of cachexia (*Figures* 2B and S2D–S2F). Increased PCs were only observed in the C26‐cx group from experiment 3 and in Apc^Min/+^ mice. Higher PE levels were only associated with the C26 cachectic phenotype. While we observed that several LPC species were reduced in the plasma of each studied cachexia model (*Figure* 1C), total measured LPC levels were only significantly lower in C26‐cx mice from experiment 2 (*Figure* 2B), suggesting rather a change in LPC quality than in quantity during CCx. Interestingly, lipids from the sphingolipid family, including SMs, CERs, HCERs and LCERs, were particularly increased in virtually every cachexia model while no alterations were observed at an earlier time point after tumour cell implantation (*Figures* 2C–2E and S2G). Notably, sphingolipids gradually rose during cachexia development as illustrated in experiment 3 on C26‐precx and C26‐cx mice (*Figures* 2C–2E). Thus, our data so far demonstrate that the elevation of circulating sphingolipid levels, a lipid class closely associated with insulin resistance, is common between different CCx mouse models and increases progressively with cachexia development.

### Ceramide metabolism is increased in livers of cachectic mice

**Figure 3:**
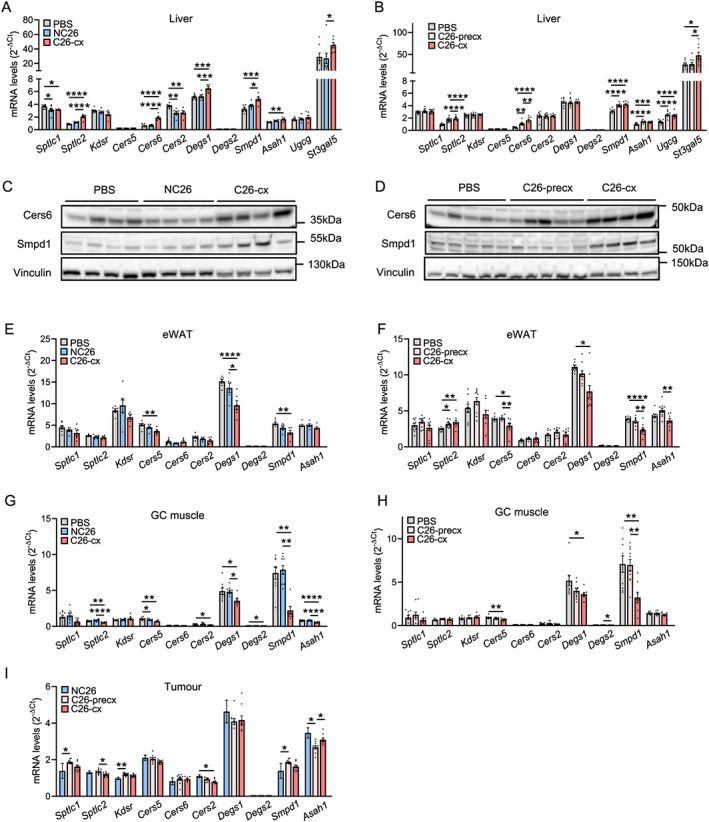
Expression of key enzymes involved in sphingolipid metabolism is altered in metabolic organs. (*A*,*B*) Liver mRNA levels of enzymes involved in ceramide metabolism in phosphate‐buffered saline (PBS) (grey bars, *n* = 8 animals), non‐cachectic NC26 (blue bars, *n* = 7 animals), and cachectic C26 (C26‐cx, red bars, *n* = 6 animals) tumour‐bearing mice (*A*, *experiment 2*); and PBS (grey bars, *n* = 10 animals), pre‐cachectic (C26‐precx, pink bars, *n* = 11 animals), and cachectic (C26‐cx, red bars, *n* = 9 animals) C26 tumour‐bearing mice (*B*, *experiment 3*). (*C*,*D*) Liver protein levels of Cers6 and Smpd1 from *experiments 2 and 3*. Vinculin was used as loading control. (*E*–*I*) mRNA levels of enzymes involved in ceramide metabolism in epididymal white adipose tissue (eWAT) (*E*,*F*), GC muscle (*G*,*H*), and tumour (*I*). Data are mean ± SEM; statistical analyses were performed using unpaired one‐way ANOVA or Kruskal–Wallis tests with Bonferroni or Dunn's *post‐hoc* tests, respectively. Tests were two sided. *P < 0.05, **P < 0.01, ***P < 0.001, ****P < 0.0001.

Liver and adipose tissue have been reported as major contributors to the circulating levels of CERs, although other metabolic organs as well as tumours are also able to synthesize CERs.^28^, ^29^, ^30^ We therefore determined mRNA expression levels of genes coding for key enzymes of the *de novo* CER synthesis pathway (serine palmitoyl transferases *Sptlc1*, *Sptlc2*; 3‐ketosphinganine reductase *Kdsr*; CER synthases *Cers5*, *Cers6*, *Cers2*; dihydroceramide desaturases *Degs1*, *Degs2*) and of the salvage pathway (i.e. sphingomyelin hydrolysis into CER by acid sphingomyelinase *Smpd1*), as well as CER degradation (*Asah1*) in metabolic organs and tumours from NC26, C26‐precx, and C26‐cx mice. *Sptlc2*, *Cers6*, *Degs1*, and *Smpd1* were strongly upregulated in livers of cachectic C26‐cx compared with PBS and NC26 control mice (*Figures* 3A and 3B). Of note, the CER synthase Cers6, which catalyzes CER synthesis from palmitate, exhibited a gradual rise during cachexia development and mirrored sphingolipid levels in C26‐precx and C26‐cx mice (*Figures* 3B and 2C‐2E). Higher expression of Cers6 and Smpd1 in livers of C26‐cx mice was confirmed by western blot analysis (*Figures* 3C‐3D and S3A‐S3B). mRNA expression of the ceramidase *Asah1* was also upregulated, suggesting an increase in whole CER turnover in livers of cachectic mice (*Figures* 3A and 3B). Enzymes responsible for HCER synthesis (*Ugcg*) and conversion of LCER into more complex glycosphingolipids (i.e. gangliosides) (*St3gal5*) were increased as well, in accordance with the lipidomics data (*Figures* 3A and 3B). The same regulations were observed in livers from LLC and Apc^Min/+^ mice (*Figures* S3C and S3D). In accordance with the unchanged lipid profile of C26‐noncx mice, liver gene expression was not affected at an early stage of the disease (*Figure* S3E). Epididymal WAT (eWAT) and GC muscle exhibited reduced expression of *Cers5*, *Degs1*, *Degs2*, and *Smpd1* in C26‐cx mice (*Figures* 3E–3H); the expression profile in the tumours of C26‐cx, C26‐precx, and NC26 mice was similar, showing only slightly unspecific alterations (*Figure* 3I). Overall, these results suggest that the liver might be the main contributor to the elevation of plasma sphingolipids during cachexia development.

### Changes in plasma lipid species in cachectic mice are consistent with the changes in cachectic cancer patients

**Figure 4:**
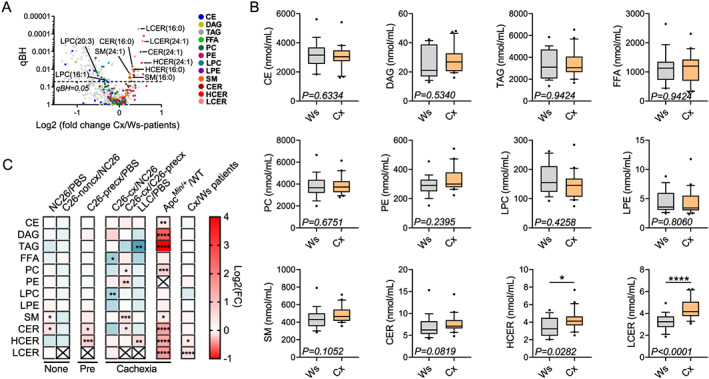
Profiles of circulating lipids in cachectic and weight‐stable cancer patients resemble changes in cancer cachexia (CCx) mouse models. (*A*) Volcano plot showing the changes in plasma lipid species between cachectic (Cx, *n* = 20 individuals) and weight stable (Ws, *n* = 19 individuals) cancer patients. The dashed line indicates the adjusted *P* value (qBH) of 0.05. Statistical analyses were performed using two‐sided Wilcoxon rank sum tests. *P* values were adjusted for multiple testing using the Benjamini–Hochberg correction method. (*B*) Plasma lipid class sum concentrations of weight stable (Ws, grey boxplots, *n* = 19 individuals) and cachectic (Cx, orange boxplots, *n* = 20 individuals) cancer patients. Data are median 10–90 percentile. (*C*) Heat map showing the fold change in plasma lipid class concentrations in non‐cachectic (NC26/PBS, C26‐noncx/NC26), pre‐cachectic (C26‐precx/PBS), and cachectic mice (C26‐cx/NC26, C26‐cx/C26‐precx, LLC/PBS, Apc^Min/+^/WT) as well as cachectic cancer patients (Cx/Ws patients). Statistical analyses were performed using unpaired *t* tests or Mann–Whitney tests (*B*,*C*) and unpaired one‐way ANOVA or Kruskal–Wallis tests with Bonferroni or Dunn's *post‐hoc* tests (*C*). Tests were two sided. *P < 0.05, **P < 0.01, ***P < 0.001, ****P < 0.0001.

**Figure 5:**
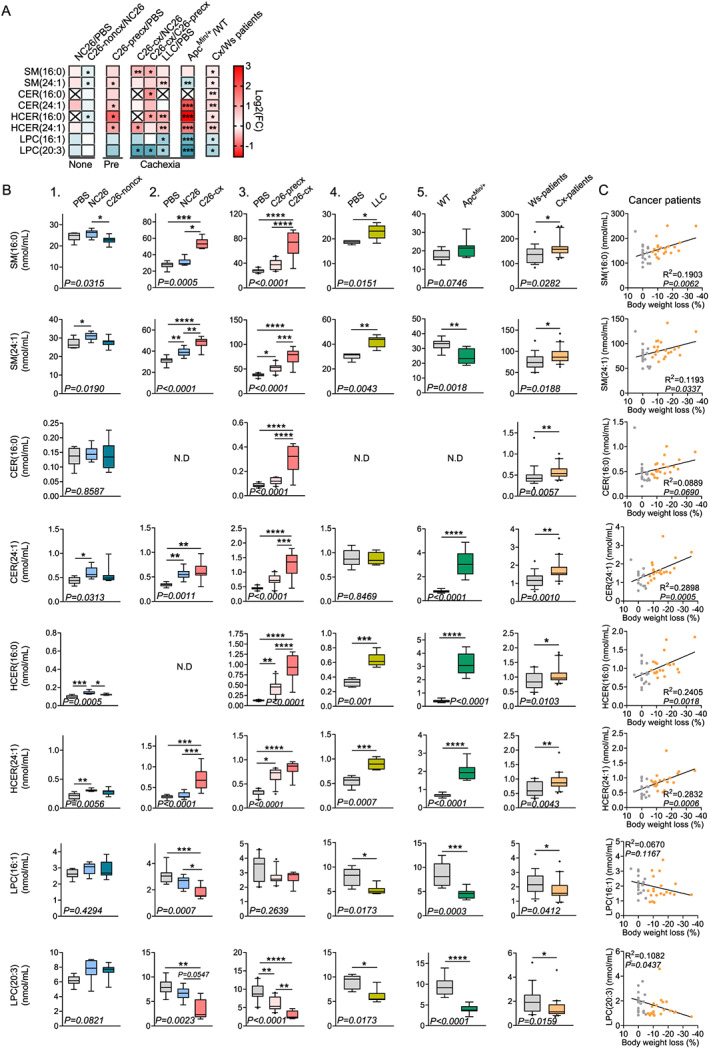
Commonly altered lipid species in mice and patients correlate with cachexia severity. (*A*) Heat map showing the fold change of the most consistently affected lipid species between non‐cachectic [NC26/phosphate buffered saline (PBS), C26‐noncx/NC26], pre‐cachectic (C26‐precx/PBS), and cachectic mice (C26‐cx/NC26, C26‐cx/C26‐precx, LLC/PBS, Apc^Min/+^/WT) as well as cachectic cancer patients (Cx/Ws patients). Statistical analyses were performed using two‐sided Wilcoxon rank sum tests. *P* values were adjusted for multiple testing using the Benjamini–Hochberg correction method. *P < 0.05, **P < 0.01, ***P < 0.001. (*B*) Plasma concentration of the top lipid species associated with cachexia. From left to right: PBS (grey boxplots, *n* = 6 animals), non‐cachectic NC26 (blue boxplots, *n* = 7 animals), and C26 (C26‐noncx, dark blue boxplots, *n* = 7 animals) tumour‐bearing mice. PBS (grey boxplots, *n* = 8 animals), non‐cachectic NC26 (blue boxplots, *n* = 9 animals), and cachectic C26 tumour‐bearing mice (C26‐cx, red boxplots *n* = 7 animals). PBS (grey boxplots, *n* = 10 animals), pre‐cachectic (C26‐precx, pink boxplots, *n* = 11 animals), and cachectic (C26‐cx, red boxplots, *n* = 9 animals) C26 tumour‐bearing mice. PBS (grey boxplots, *n* = 5 animals) and cachectic LLC tumour‐bearing mice (yellow boxplots, *n* = 6 animals). Wildtype (WT, grey boxplots, *n* = 9 animals) and cachectic Apc^Min/+^ mutant mice (green boxplots, *n* = 9 animals). Weight stable (Ws, grey boxplots, *n* = 19 individuals) and cachectic (Cx, orange boxplots, *n* = 20 individuals) cancer patients. (*C*) Correlations between the concentration of lipid species and percentage of body weight loss in weight stable (grey dots, *n* = 19 individuals) and cachectic (orange dots, *n* = 20 individuals) cancer patients. Data are median 10–90 percentile (*B*). Statistical analyses were performed using unpaired one‐way ANOVA or Kruskal–Wallis tests with Bonferroni or Dunn's *post‐hoc* tests (*B, experiments 1‐3*), unpaired *t* test or Mann‐Whitney test (*B, experiments 4‐5 and cancer patients*) and linear regression (*C*). Tests were two sided. *P < 0.05, **P < 0.01, ***P < 0.001, ****P < 0.0001.

To confirm the results observed in our different mouse models, we next performed lipidomic analyses on plasma from cachectic and weight stable patients with gastrointestinal cancers. Cachectic cancer patients were characterized by a mean body weight loss of 15% vs. 1% for the weight stable patients, low haemoglobin and high plasma C‐reactive protein (CRP) levels (*Table* 1). Circulating amounts of most of the lipid classes remained unchanged between cachectic and weight‐stable patients. However, higher circulating levels of sphingolipids and decreased levels of some LPC species were associated with cachexia in humans (*Figures* 4A and 4B). Strikingly, a significant elevation of HCER and LCER levels and a trend towards increased total SM and CER levels were common features between our different mouse models of cachexia and the cachectic patients (*Figures* 4B and 4C). Furthermore, cachectic patients also exhibited lower levels of several TAG species (*Figure* 4A). Concerning individual lipid species, 11 were mutually elevated, and 70 were mutually reduced between at least one CCx mouse model and cachectic patients in comparison with non‐cachectic mice and weight‐stable cancer patients, respectively. Among the upregulated species, six were regulated consistently in different mouse models and patients. They belonged to the sphingolipid family, namely, SM(16:0), SM(24:1), CER(16:0), CER(24:1), HCER(16:0), and HCER(24:1) (*Figures* 5A–5B and S4A‐S4B). Among the downregulated species, TAGs represented almost all of the affected lipids and were only negatively regulated in patients and LLC mice, as depicted in the heat maps in *Figures* S4C–S4I. Of note, TAGs containing FA(18:3) and FA(20:5), potentially corresponding to the essential FAs alpha‐linolenic acid (18:3) and eicosapentaenoic acid (20:5), were the most affected ones in cachectic patients. We identified LPC(16:1) and LPC(20:3) as the most consistently reduced lipid species during cachexia development among the different mouse models and cachectic patients (*Figures* 5A‐5B and S4J). Remarkably, the circulating levels of the eight identified lipid species, which were regulated in the same manner in all mouse models and patients, were closely correlated with the percentage of body weight loss both in mice and humans (*Figures* 5C and S5). Alterations in circulating cytokine levels and cytokine ratios are known hallmarks of CCx. Interestingly, these alterations correlated with the degree of body weight loss in the studied patient cohort (*Table* 2). Furthermore, some of the identified hit lipid species were also associated with IL6 and IL1β levels as well as various ratios of circulating cytokines (TNFα/IL10, TNFα/IL8, and IL6/IL1β) (*Table* 2). Accordingly, none of these lipid species were altered in C26‐noncx mice (*Figures* 5A‐5B and S5), confirming their close association with cachexia and implying a potential functional connection with processes contributing to weight loss. Furthermore, several of these hit lipids rose sequentially across the pre‐cachectic and cachectic stages, supporting a potential role as early prognostic markers of cachexia.

**Table 1:** Human clinical data

	**Weight stable cancer**	**Cancer cachexia**	**P value**
**Clinical Parameters**			
Overall (n)	19	20	
Female/Male (n)	9/10	10/10	
Age (years)	57.42 ± 2.58	59.95 ± 3.57	0.5733
BW loss (%)	‐1.08 ± 0.56	‐14.96 ± 1.62	<0.0001
BMI (kg/m^2^)	25.59 ± 1.62	20.75 ± 0.68	<0.0001
**Tumor site**			
Colon and rectum	17 (89.5 %)	16 (80.0 %)	
Stomach	2 (10.5 %)	3 (15.0 %)	
Adenocarcinoma	0 (0.0 %)	1 (5.0 %)	
**Tumor staging**			
I	5	3	
IIA/IIB/IIC	7	4	
**I/II**	**12**	**7**	
IIIA/IIIB/IIIC	3	7	
IVA/IVB	1	2	
**III/IV**	**4**	**9**	
**Non‐classified**	**3**	**4**	
**Serum Parameters**			
Haemoglobin (g/dL)	12.93 ± 0.39	11.17 ± 0.6640	0.0280
CRP (mg/L)	5.56 ± 1.12	9.20 ± 0.8587	0.0132
Albumin (g/dL)	3.522 ± 0.12	3.308 ± 0.08869	0.1566
Cholesterol (mg/dL)	187.4 ± 13.61	199.4 ± 11.54	0.5005
HDL (mg/dL)	43.06 ± 4.30	43.90 ± 3.22	0.8745
LDL (mg/dL)	110.6 ± 9.34	109.9 ± 8.21	0.9513
Triacylglycerols (mg/dL)	163.4 ± 18.73	165.4 ± 15.04	0.4472

BMI, body mass index; BW, body weight; CRP, C‐reactive protein; HDL, high‐density lipoprotein; LDL, low‐density lipoprotein.

Data are mean ± SEM. Statistical analyses were performed using unpaired *t* test or Mann–Whitney test. Tests were two‐sided.

**Table 2:** Associations of lipid species with circulating cytokine levels and cytokine ratios in cancer patients

Lipid species	IL1β	IL6	TNFα/IL10	TNFα/IL8	IL6/IL1β	IL6/IL8
	*R* ^2^	*R* ^2^	*R* ^2^	*R* ^2^	*R* ^2^	*R* ^2^
SM(16:0)	0.0143	0.1559 (*trend*)	0.0470	0.0006	0.2374*	0.0382
SM(24:1)	0.0047	0.1349	0.0447	0.0001	0.2592*	0.0354
CER(16:0)	0.0009	0.0835	0.2789*	0.0475	0.1274	0.1130
CER(24:1)	0.01082	0.2036*	0.0092	0.0065	0.2894*	0.1658 (*trend*)
HCER(16:0)	0.2462*	0.4853***	0.0656	0.2189*	0.2402*	0.0451
HCER(24:1)	0.3144***	0.4851***	0.1026	0.2379*	0.2209*	0.1953 (*trend*)
LPC(16:1)	0.0002	0.0306	0.2284*	0.3218**	0.0186	0.1481 (*trend*)
LPC(20:3)	0.0342	0.1346	0.5835***	0.4252**	0.1307	0.0933
Body weight loss (%)	0.0438	0.3482**	0.1996*	0.2087*	0.3035**	0.2722*

^*^ *P* < 0.05,

^**^ *P* < 0.01,

^***^ *P* < 0.001.

CER, ceramide; HCER, hexosyl‐ceramide; LPC, lysophosphatidylcholine; SM, sphingomyelin.

Statistical analyses were performed using simple linear regression. Associations are illustrated by *R* ^2^ values. *Trend* means 0.05 < *P* < 0.1.

## Discussion

**Figure 6:**
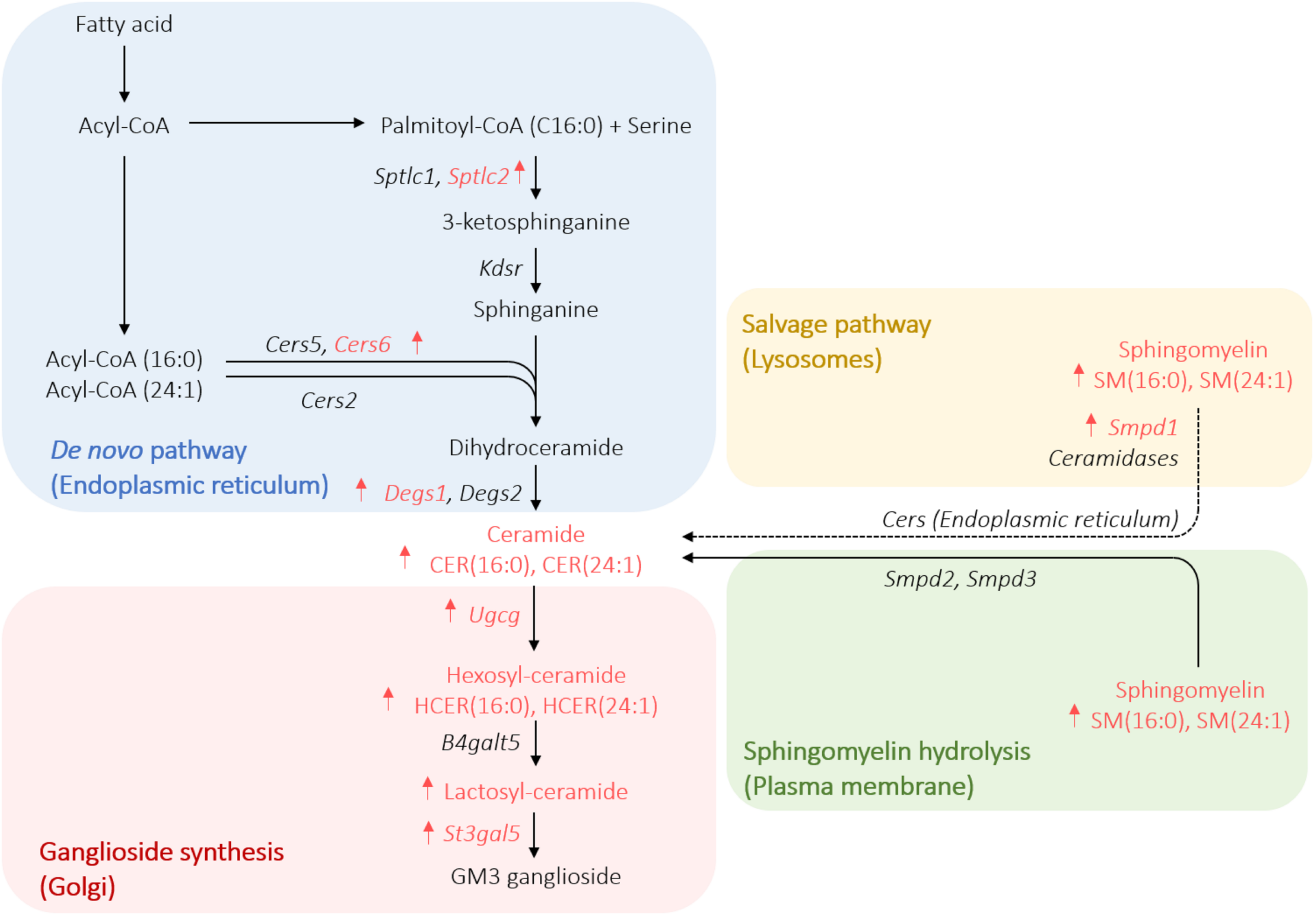
Overview of the main ceramide synthesis enzymes altered in livers of cachectic mice, as well as the main sphingolipid species increased with cancer cachexia.

Cancer cachexia (CCx) is a multifactorial syndrome that affects a large number of cancer patients, contributing to high mortality rates and poor quality of life.^3^ Although it has already been shown that bioactive lipids are altered upon cancer incidence,^28^, ^31^, ^32^, ^33^, ^34^ there are still many unanswered questions concerning circulating lipid alterations in CCx. This study is the first to report a broad lipidomic screen, using the Lipidyzer™ platform, to identify specific lipid species as markers of CCx by comparing plasma from different CCx mouse models (NC26, C26, and LLC cell transplantation, Apc ^Min/+^ mutation), and cachectic and weight stable cancer patients, which aimed to address whether the results found in animal models were translatable. We observed a clear discrimination between the lipid profiles of cachectic tumour‐bearing mice and non‐cachectic tumour‐bearing or PBS control mice. Decreases in many LPC species and increases in numerous sphingolipids, including SMs, CERs, HCERs, and LCERs, were the most consistent features observed during cachexia in both mice and humans. We identified eight lipid species that were regulated in a similar manner between the different mouse models and patient groups. Six of them, namely SM(16:0), SM(24:1), CER(16:0), CER(24:1), HCER(16:0), and HCER(24:1) were increased, while LPC(16:1) and LPC(20:3) were decreased during cachexia. The circulating amounts of these eight lipid species closely correlated with the percentage of body weight loss in mice and humans, implying a potential functional role in processes contributing to weight loss. Increased circulating sphingolipid levels were associated with higher expression of enzymes responsible for CER synthesis in livers of cachectic mice (*Figure* 6).

Decreases in LPC species, notably LPC(20:3), have already been reported in cachectic cancer patients and have been associated with weight loss and inflammatory markers,^14^, ^16^ supporting the strength of our data. Contrasting results on LPC reported in a recent study^35^ may relate to differences in the sample preparation procedure, as this group performed a single‐step deproteinization using acetonitrile precipitation while we preferred a liquid–liquid MTBE extraction of lipids using organic solvent, one of the standard methods for lipidomics.^36^ Consistent with our findings, decreased LPC levels have also been correlated with mortality in sepsis patients, another disease often associated with cachexia.^37^, ^38^, ^39^ Additionally, Semba *et al*. have recently shown that low plasma LPC levels in humans, particularly LPC(16:1) and LPC(20:3), are associated with impaired mitochondrial oxidative capacity in skeletal muscle during ageing.^40^ This dysregulation was also shown to be involved in muscle atrophy of tumour‐bearing animals; however, this has yet to be confirmed in cancer patients.^41^

In the present study, TAG species containing FA (18:3) and FA (20:5) in particular, were reduced in the plasma of cachectic cancer patients. This is in accordance with a previous report^15^ in which decreased circulating levels of polyunsaturated FAs, especially n‐3 FFAs, were found in cachectic patients while decreased n‐6 FFAs were observed in all cancer patients (Ws and Cx). In the same study, cachexia was associated with an increase in the saturated to unsaturated FA ratio in the circulation. These alterations are likely to contribute to the inflammatory status in cachexia, as the presence of higher saturated FA content and diminished n‐3:n‐6 FA ratio are known to influence the onset, progression, and outcome of chronic diseases. The reduction of these two FAs could potentially be related to a deficiency in the essential omega‐3 FAs alpha‐linolenic acid (18:3) and eicosapentaenoic acid (20:5). These observations support the therapeutic approaches of omega‐3 eicosapentaenoic acid (EPA) and docosahexaenoic acid (DHA) supplementation in cachectic cancer patients. Based on their anti‐inflammatory properties and potential direct effect on skeletal muscle, one could hypothesize that a depletion in these essential FAs would exacerbate inflammation and tissue wasting in cancer patients.^42^ Various clinical studies have been conducted to assess the potential effects of omega‐3 FA supplementation in cachexia, all summarized in a recent systematic review, which selected seven clinical studies based on the quality of their methodology.^43^ Interestingly, only one study, conducted on pre‐cachectic cancer patients, showed statistically and clinically positive results of EPA and DHA supplementation.^44^, ^45^ The intervention group exhibited improved weight maintenance, superior preservation of fat free mass, as well as increased global health status, physical, cognitive and social functions, and an overall improved quality of life. Other studies including cachectic cancer patients did not show significant body weight enhancement upon omega‐3 FA supplementation.^46^, ^47^, ^48^ However, these studies still reported improvement in energy expenditure and physical activity,^48^ as well as mild increases in body weight, lean body mass, and physical function in a subgroup of patients treated with 2 g of EPA per day.^46^ These data suggest that omega‐3 FA supplementation might be more beneficial for pre‐cachectic patients with a less advanced disease stage and lower malnutrition, than for patients with already established cachexia.^49^ Of note in the current study, depletion in essential omega‐3 FAs was exclusively observed in cachectic cancer patients while the different mouse models of CCx exhibited very different TAG profiles. This may explain why some EPA and fish oil supplementation studies in C26 tumour‐bearing mice have been unsuccessful in rescuing body weight loss and organ wasting in the past.^50^, ^51^ Nevertheless, the greater similarity between the TAG profile of cachectic patients and LLC tumour‐bearing mice indicates that this model seems to be the most suitable for future preclinical mechanistic studies in the field.

Most importantly, a robust increase in circulating sphingolipid levels, notably CERs, was a common feature of CCx between our different cachexia mouse models and cachectic cancer patients. This may be the consequence of an increased CER turnover in the liver during cachexia development, while expression of CER‐related metabolic enzymes in tumour, skeletal muscle, and adipose tissue were not affected or even downregulated. CER metabolism has already been associated with cancer, as shown in a cohort of patients suffering from breast cancer. The authors demonstrated that a high expression of enzymes involved in the different CER synthesis pathways was associated with unfavourable prognosis.^29^ Cachexia was however not studied in this context. High CER levels potentially contribute to tissue wasting in different ways. First, increased expression of enzymes responsible for CER synthesis and degradation in the liver might represent a futile cycle contributing to energy wasting in CCx. Second, CERs can disrupt liver metabolism by stimulating FA uptake and steatosis.^30^, ^52^, ^53^ This could in part contribute to the liver steatosis observed in cachexia, a process still incompletely characterized. In addition, liver‐derived CERs are major actors in the development of obesity‐induced insulin resistance.^52^, ^53^ Through activation of protein phosphatase 2A and atypical protein kinase Cζ, CERs directly interfere with insulin signalling in metabolic organs such as liver, adipose tissue and skeletal muscle.^8^, ^54^ Additionally, HCERs and LCERs are precursors of a more complex class of glycosphingolipids, named gangliosides, which directly impair insulin receptor function.^8^, ^55^ Insulin resistance has previously been shown to be a common feature of cachectic cancer patients and mouse models, potentially contributing to the muscle wasting observed in CCx. Therefore, the increase in circulating CERs observed in our study as well as CER accumulation in metabolic tissues could in part be responsible for the resistance to anabolic insulin signalling in CCx, thereby worsening tissue wasting.^56^ Accordingly, CERs induced L6 myotube atrophy *in vitro*.^57^ *In vivo*, treatment of C26 tumour‐bearing mice with myriocin, an inhibitor of the *de novo* CER synthesis pathway, partially prevented induction of atrophy markers and skeletal muscle loss.^57^ However, myriocin treatment did not efficiently reduce muscular CER content in this study. This might be due to incomplete inhibition of CER synthesis as our data suggest that the salvage pathway is also activated in cachexia. A combinatorial treatment using both serine palmitoyl transferase (i.e. myriocin) and acid sphingomyelinase (i.e. desipramine) inhibitors must be of interest to overcome this problem. Interestingly, treatment with desipramine alone showed a protective effect on liver function and fibrosis and prolonged survival of different mouse models of sepsis.^58^, ^59^

As the timing is very important for the success of a given therapy, treatment should be initiated as early as possible, emphasizing the need for biomarkers in the context of CCx. High circulating sphingolipid levels have been associated with many pathologies and have already been proposed as biomarkers for different diseases.^28^, ^33^ Notably, increased circulating levels of CER(16:0) or CER(24:1) in humans have been observed in ovarian cancer, advanced stage colorectal cancer, autoimmune diseases such as multiple sclerosis, type 2 diabetes, Alzheimer's disease, sepsis, as well as cardiovascular diseases.^28^, ^33^, ^37^ High HCER(16:0) and HCER(24:1) levels have also been reported in patients with multiple sclerosis.^28^ In sepsis, a rise in CERs has been associated with higher mortality in patients.^37^ Additionally, SM(16:0), CER(16:0), and CER(24:1) were among the lipid species most strongly associated with mortality in a cohort of patients suffering from cardiovascular diseases.^60^ In our study, SM, CER, and HCER (16:0) and (24:1) were the most consistently affected lipid species in CCx and strongly correlated with body weight loss in mice and humans. Some of these lipid species are also associated with altered profile of cytokines in cachexia. Interestingly, the change in these species was progressive during the time course of cachexia development with no or minor modifications at an early stage of the disease and an intermediate profile in pre‐cachectic C26 tumour‐bearing mice. This would support using sphingolipids as potential biomarkers for CCx, even prior to significant body weight loss. However, future prospective clinical studies on cancer patients would be necessary to address this point.

Taken together, this work demonstrates the identification of specific, common circulating lipid species as markers of murine and human CCx. Changes in several lipid species, mainly sphingolipids, have been associated with cachexia severity (i.e. body weight loss) and potentially contribute to tissue wasting. Furthermore, their role as potential biomarkers was discussed, the most noteworthy aspect being that alterations could be precociously detected in regard to SM, CER, and HCER (16:0) and (24:1), hence implying a potential application in the diagnosis of early cachexia. Nevertheless, future studies are necessary to validate the possible uses and limitations of sphingolipid species as prognostic markers and treatment targets in CCx.

## Conflict of interest

All authors declare that they have no conflict of interest.

## Funding

This work was supported by the Helmholtz Alliance ‘Ageing and Metabolic Programming’ (AMPro) to J. Z and M.R.. P.M., M.B.D. and S.H. are supported by the German Research Foundation (*Deutsche Forschungsgemeinschaft*, SFB1321). D.K. is supported by an Erwin Schrödinger Fellowship from the Austrian Science Fund (FWF, J4224 – B34). S.F.S. is supported by a fellowship from the Novo Nordisk Foundation (NNFOC150019050). Sampling of human specimen was funded by FAPESP 2012/50079‐0 to M.S.

## Supporting information


**Figure S1.** (A) Body weight loss (tumour subtracted) expressed as percentage of initial (*experiments 1–4*) or maximal body weight (*experiment 5*). (B) Tumour weight. (C‐D) Inguinal (iWAT) (C) and epididymal (eWAT) (D) white adipose tissue weights. (E‐F) GC muscles (E) and heart (F) weights. From left to right: PBS (grey, *n* = 6 animals), non‐cachectic NC26 (blue, *n* = 7 animals) and C26 (C26‐noncx, dark blue, n = 7 animals) tumour‐bearing mice. PBS (grey, *n* = 8 animals), non‐cachectic NC26 (blue, *n* = 9 animals) and cachectic C26 tumour‐bearing mice (C26‐cx, red, *n* = 7 animals). PBS (grey, *n* = 10 animals), pre‐cachectic (C26‐precx, pink, *n* = 11 animals) and cachectic (C26‐cx, red, *n* = 9 animals) C26 tumour‐bearing mice. PBS (grey, *n* = 5 animals) and cachectic LLC tumour‐bearing mice (yellow, *n* = 6 animals). Wildtype (WT, grey, n = 9 animals) and cachectic Apc^Min/+^ mutant mice (green, n = 9 animals). Data are mean ± SEM Statistical analyses were performed using unpaired one‐way ANOVA or Kruskal‐Wallis tests with Bonferroni or Dunn's post‐hoc tests respectively (*experiments 1–3*) and unpaired *t* test or Mann–Whitney test (*experiments 4–5* and tumour). Tests were two sided. * *p* < 0.05, **p < 0.01, ***p < 0.001, ****p < 0.0001.
**Figure S2.** (A‐G) Plasma lipid class sum concentrations for each experiment. CE (A), DAG (B), FFA (C), PC (D), PE (E), LPE (F), LCER (G). From left to right: PBS (grey boxplots, *n* = 6 animals), non‐cachectic NC26 (blue boxplots, *n* = 7 animals) and C26 (C26‐noncx, dark blue boxplots, n = 7 animals) tumour‐bearing mice. PBS (grey boxplots, *n* = 8 animals), non‐cachectic NC26 (blue boxplots, *n* = 9 animals) and cachectic C26 tumour‐bearing mice (C26‐cx, red boxplots n = 7 animals). PBS (grey boxplots, *n* = 10 animals), pre‐cachectic (C26‐precx, pink boxplots, *n* = 11 animals) and cachectic (C26‐cx, red boxplots, *n* = 9 animals) C26 tumour‐bearing mice. PBS (grey boxplots, *n* = 5 animals) and cachectic LLC tumour‐bearing mice (yellow boxplots, *n* = 6 animals). Wildtype (WT, grey boxplots, n = 9 animals) and cachectic Apc^Min/+^ mutant mice (green boxplots, n = 9 animals).
Data are median 10–90 percentile. Statistical analyses were performed using unpaired one‐way ANOVA or Kruskal‐Wallis tests with Bonferroni or Dunn's post‐hoc tests respectively (*experiments 1–3*) and unpaired *t* test or Mann–Whitney test (*experiments 4–5*). Tests were two sided. *p < 0.05, **p < 0.01, ***p < 0.001, ****p < 0.0001.
**Figure S3.** (A‐B) Cers6 and Smpd1 protein levels in livers of PBS (grey bars, *n* = 7 animals), non‐cachectic NC26 (blue bars, *n* = 9 animals) and cachectic C26 (C26‐cx, red bars, n = 7 animals) tumour‐bearing mice (A, *experiment 2*), and PBS (grey bars, *n* = 10 animals), pre‐cachectic (C26‐precx, pink bars, n = 9 animals) and cachectic (C26‐cx, red bars, *n* = 8 animals) C26 tumour‐bearing mice (B, *experiment 3*). Vinculin was used as loading control.
(C‐E) Liver mRNA levels of enzymes involved in ceramide metabolism. PBS (grey bars *n* = 4 animals) and LLC tumour‐bearing mice (yellow bars, n = 8 animals) (C, *experiment 4*); wild type (grey bars, *n* = 9 animals) and Apc^Min/+^ mutant mice (green bars, n = 9 animals) (D, *experiment 5*); PBS (grey bars, *n* = 6 animals), non‐cachectic NC26 (blue bars, *n* = 7 animals) and C26 (C26‐noncx, dark blue bars, n = 7 animals) tumour‐bearing mice (E, *experiment 1*).
Data are mean ± SEM Statistical analyses were performed using unpaired one‐way ANOVA or Kruskal‐Wallis tests with Bonferroni or Dunn's post‐hoc tests respectively (A‐B, E) and unpaired *t* test or Mann–Whitney test (C‐D). Tests were two‐sided. *p < 0.05, **p < 0.01, ***p < 0.001, ****p < 0.0001.
**Figure S4.** (A‐B) Heatmaps showing the fold change of SM (A), CER, HCER and LCER (B) species in non‐cachectic (NC26/PBS, C26‐noncx/NC26), pre‐cachectic (C26‐precx/PBS) and cachectic mice (C26‐cx/NC26, C26‐cx/C26‐precx, LLC/PBS, Apc^Min/+^/WT) as well as cachectic cancer patients (Cx/Ws patients). (C‐I) Heatmaps showing the fold change of TAG species carrying a FA with 0 (C), 1 (D), 2 (E), 3 (F), 4 (G), 5 (H) and 6 (I) unsaturations significantly modified in cachectic cancer patients. (J) Heatmap showing the fold change of LPC species. Statistical analyses were performed using two‐sided Wilcoxon rank sum tests. *P* values were adjusted for multiple testing using the Benjamini‐Hochberg correction method. *p < 0.05, **p < 0.01, ***p < 0.001.
**Figure S5.** Correlations between the amount of the lipid species SM(16:0), SM(24:1), CER(16:0), CER(24:1), HCER(16:0), HCER(24:1), LPC(16:1), LPC(20:3) and the percentage of body weight loss in the different mouse models. Statistical analyses were performed using linear regression.

**Table S1.** Plasma levels of cholesterol, HDL and LDL cholesterols, glucose and triacylglycerols in the different mouse experiments. Data are mean ± SEM Statistical analyses were performed using unpaired one‐way ANOVA or Kruskal‐Wallis tests with Bonferroni or Dunn's post‐hoc tests respectively (*experiments 1–3*) and unpaired *t* test (*experiments 4–5*). Tests were two‐sided. * *p* < 0.05, **p < 0.01, ***p < 0.001, ****p < 0.0001 compared to PBS or WT mice. $$p < 0.01, $$$$p < 0.0001 compared to NC26 or C26‐precx mice.
