# Supplementary figures and images for: High levels of modified ceramides are a defining feature of murine and human cancer cachexia
Source: J Cachexia Sarcopenia Muscle. 2020 Oct 8;11(6):1459–75. doi: 10.1002/jcsm.12626 (PMC7749558; doi:10.1002/jcsm.12626)

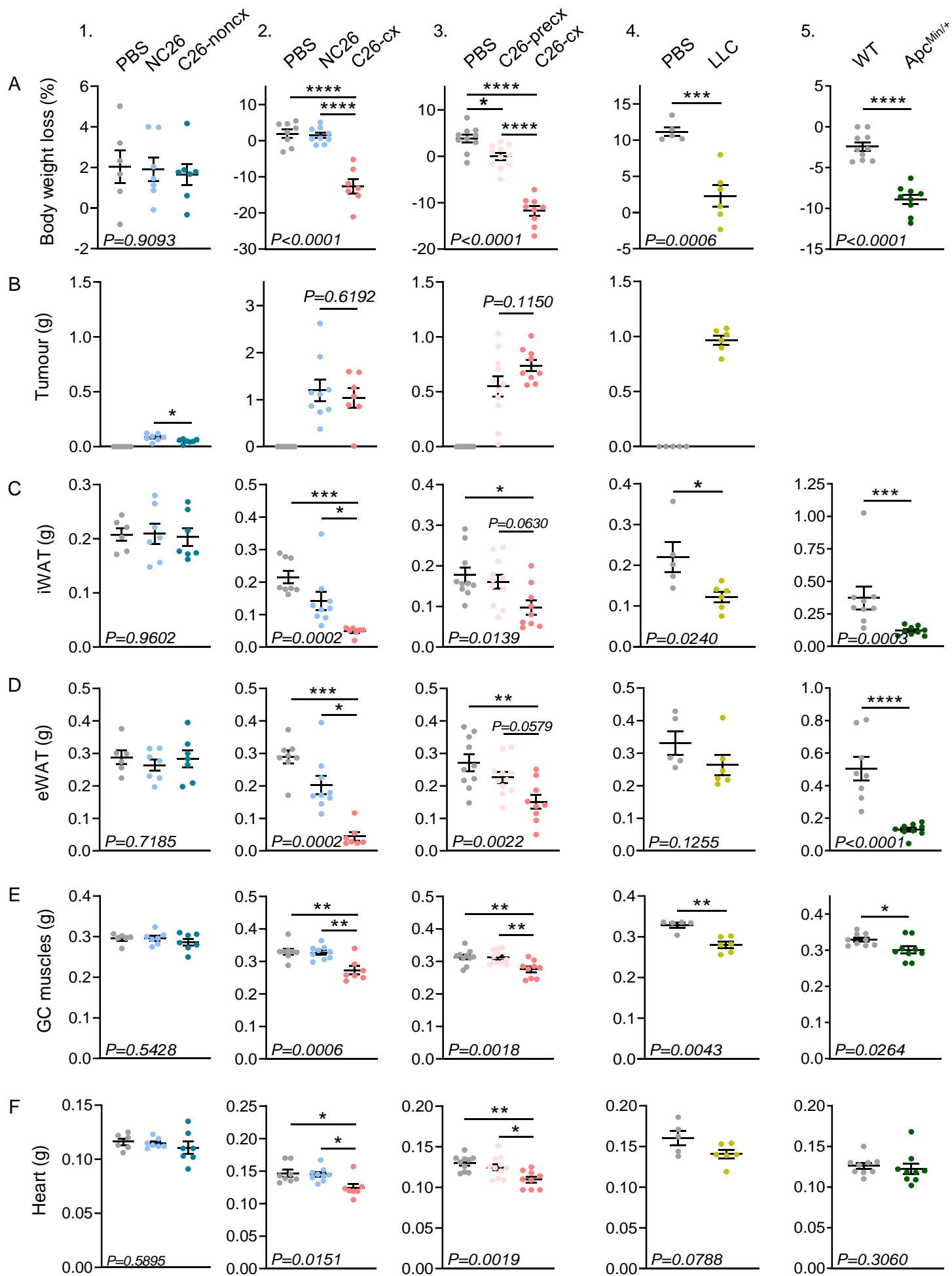

Figure S1

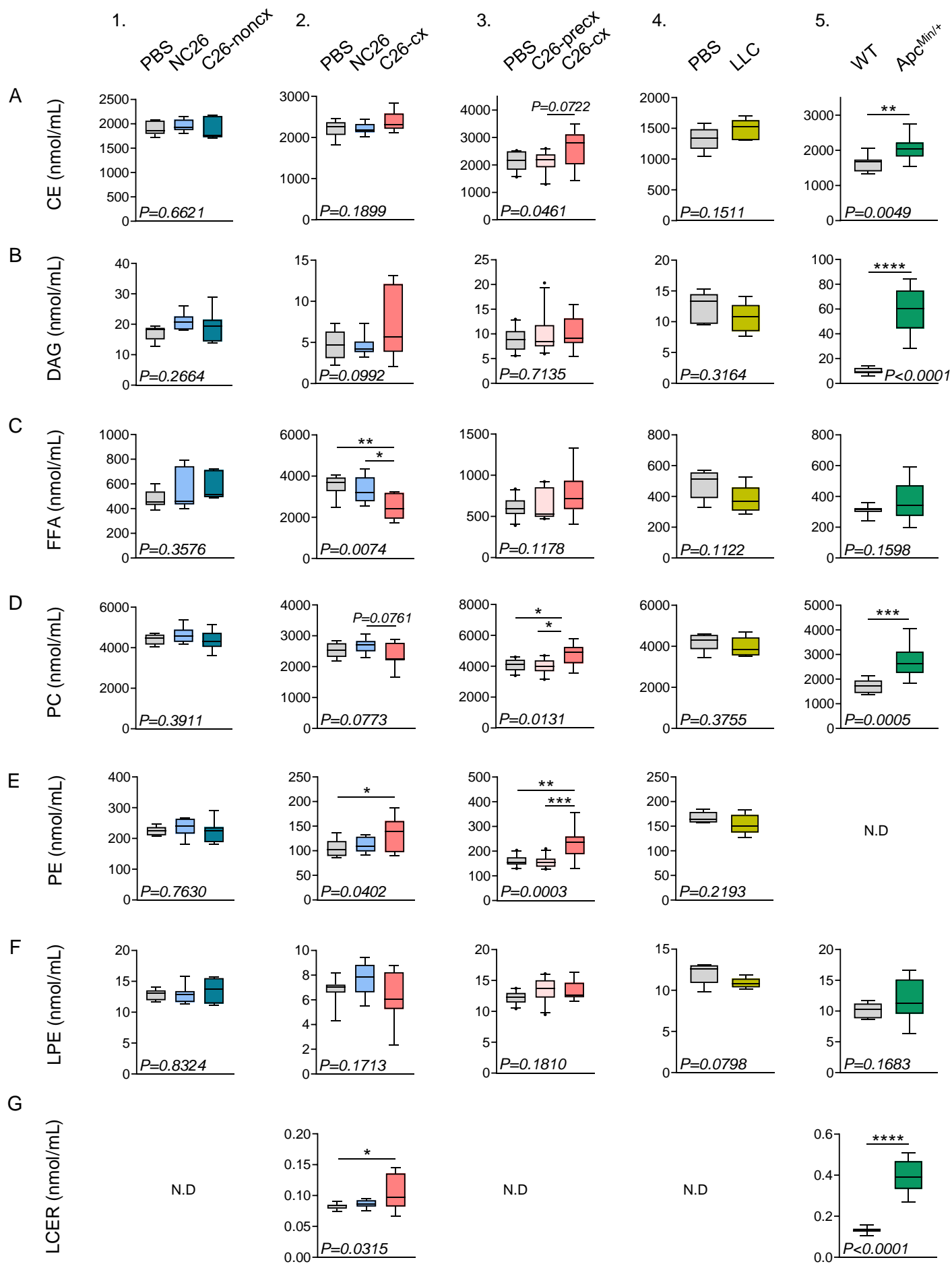

Figure S2

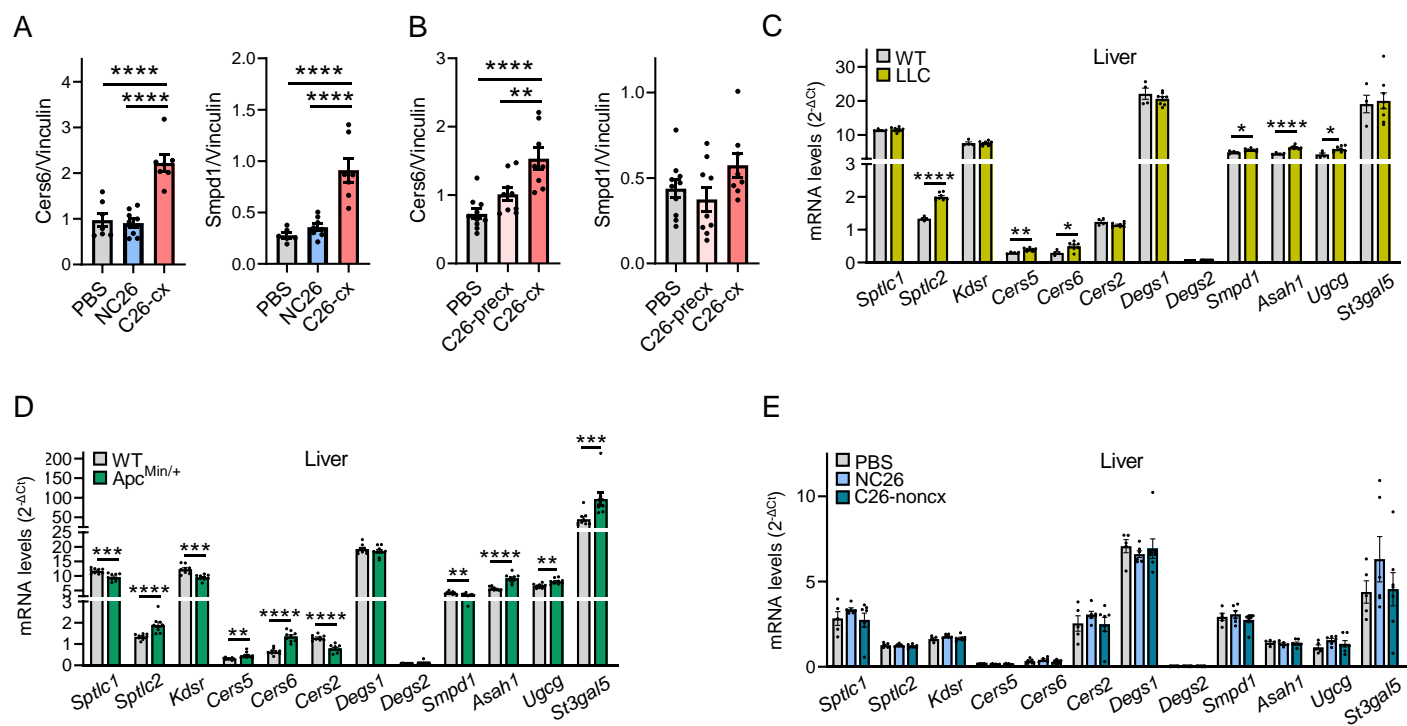

Figure S3

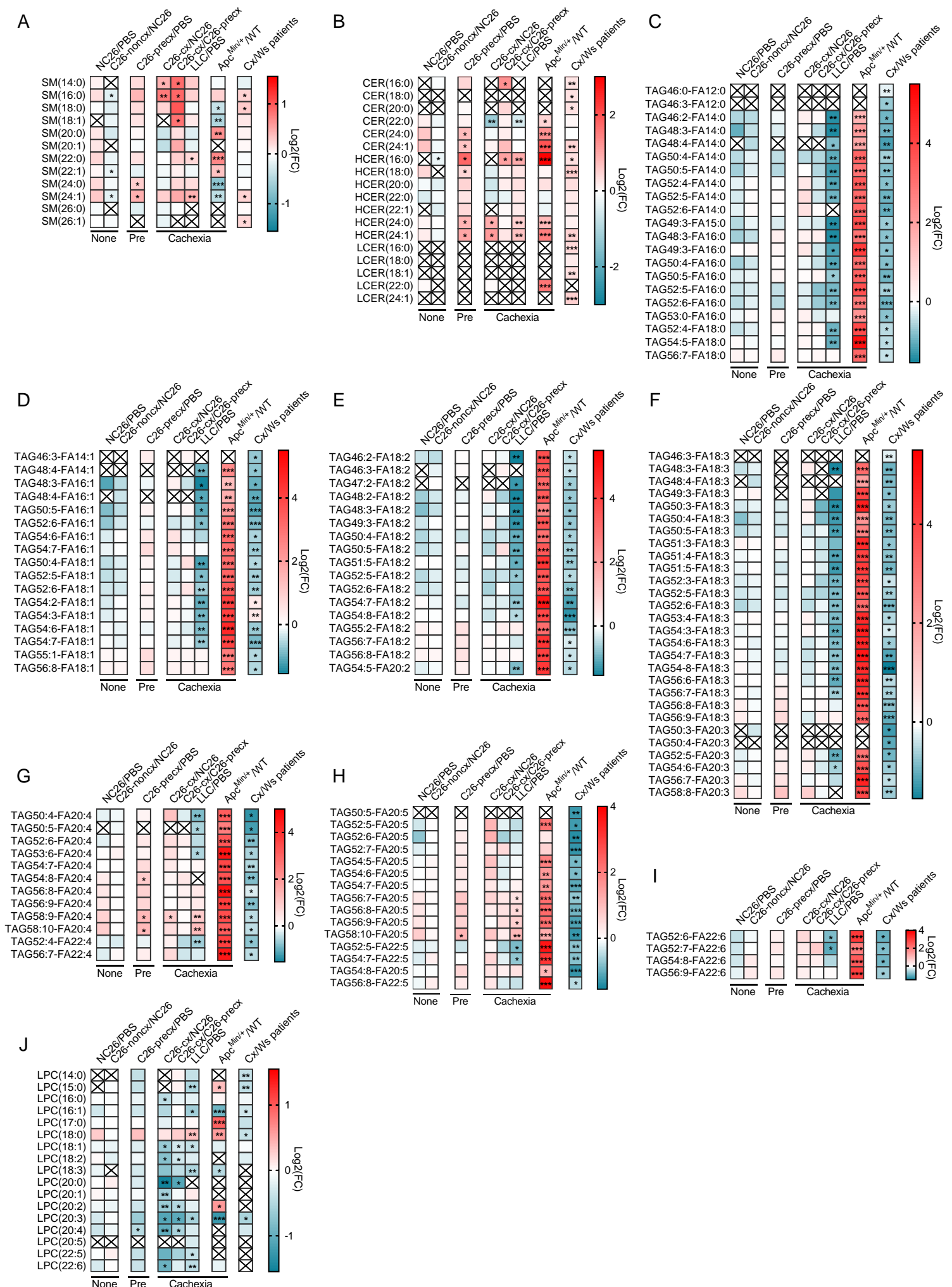

Figure S4

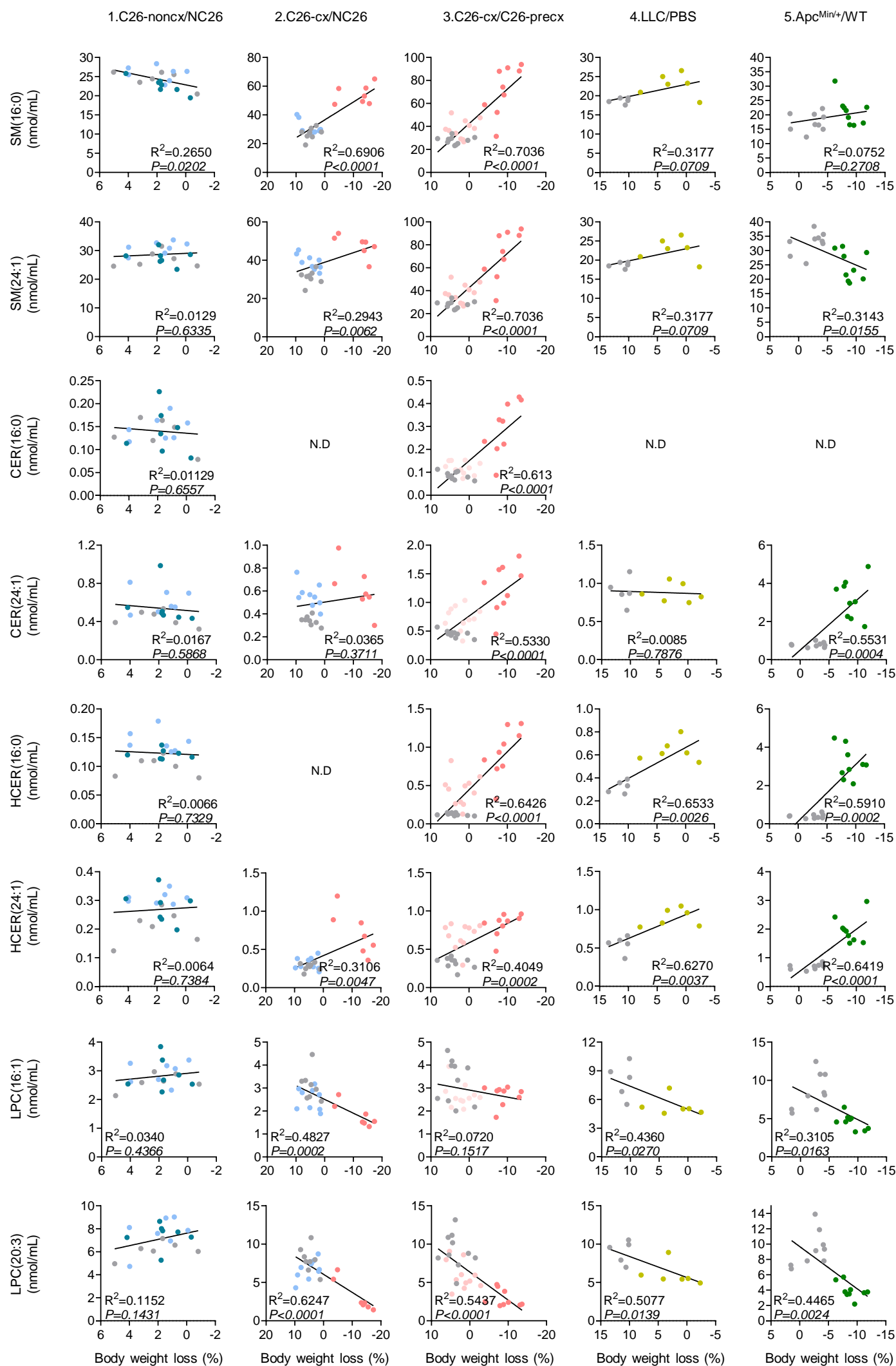

Figure S5

Supplement: Supplementary file 1 — Figure S1. (A) Body weight loss (tumour subtracted) expressed as percentage of initial (experiments 1–4) or maximal body weight (experiment 5). (B) Tumour weight. (C‐D) Inguinal (iWAT) (C) and epididymal (eWAT) (D) white adipose tissue weights. (E‐F) GC muscles (E) and heart (F) weights. From left to right: PBS (grey, n = 6 animals), non‐cachectic NC26 (blue, n = 7 animals) and C26 (C26‐noncx, dark blue, n = 7 animals) tumour‐bearing mice. PBS (grey, n = 8 animals), non‐cachectic NC26 (blue, n = 9 animals) and cachectic C26 tumour‐bearing mice (C26‐cx, red, n = 7 animals). PBS (grey, n = 10 animals), pre‐cachectic (C26‐precx, pink, n = 11 animals) and cachectic (C26‐cx, red, n = 9 animals) C26 tumour‐bearing mice. PBS (grey, n = 5 animals) and cachectic LLC tumour‐bearing mice (yellow, n = 6 animals). Wildtype (WT, grey, n = 9 animals) and cachectic ApcMin/+ mutant mice (green, n = 9 animals). Data are mean ± SEM Statistical analyses were performed using unpaired one‐way ANOVA or Kruskal‐Wallis tests with Bonferroni or Dunn's post‐hoc tests respectively (experiments 1–3) and unpaired t test or Mann–Whitney test (experiments 4–5 and tumour). Tests were two sided. * p < 0.05, **p < 0.01, ***p < 0.001, ****p < 0.0001. Figure S2. (A‐G) Plasma lipid class sum concentrations for each experiment. CE (A), DAG (B), FFA (C), PC (D), PE (E), LPE (F), LCER (G). From left to right: PBS (grey boxplots, n = 6 animals), non‐cachectic NC26 (blue boxplots, n = 7 animals) and C26 (C26‐noncx, dark blue boxplots, n = 7 animals) tumour‐bearing mice. PBS (grey boxplots, n = 8 animals), non‐cachectic NC26 (blue boxplots, n = 9 animals) and cachectic C26 tumour‐bearing mice (C26‐cx, red boxplots n = 7 animals). PBS (grey boxplots, n = 10 animals), pre‐cachectic (C26‐precx, pink boxplots, n = 11 animals) and cachectic (C26‐cx, red boxplots, n = 9 animals) C26 tumour‐bearing mice. PBS (grey boxplots, n = 5 animals) and cachectic LLC tumour‐bearing mice (yellow boxplots, n = 6 animals). Wildty [file JCSM-11-1459-s001.pdf]
