# Supplementary material for: High levels of modified ceramides are a defining feature of murine and human cancer cachexia
Source: J Cachexia Sarcopenia Muscle. 2020 Oct 8;11(6):1459–75. doi: 10.1002/jcsm.12626 (PMC7749558; doi:10.1002/jcsm.12626)
Supplement: Supplementary file 2 — Table S1. Plasma levels of cholesterol, HDL and LDL cholesterols, glucose and triacylglycerols in the different mouse experiments. Data are mean ± SEM Statistical analyses were performed using unpaired one‐way ANOVA or Kruskal‐Wallis tests with Bonferroni or Dunn's post‐hoc tests respectively (experiments 1–3) and unpaired t test (experiments 4–5). Tests were two‐sided. * p < 0.05, **p < 0.01, ***p < 0.001, ****p < 0.0001 compared to PBS or WT mice. $$p < 0.01, $$$$p < 0.0001 compared to NC26 or C26‐precx mice. [file JCSM-11-1459-s002.pdf]

Table S1.

Experiment	n	Cholesterol (mg/dL)	HDL (mg/dL)	LDL (mg/dL)	Glucose (mg/dL)	Triacylglycerols (mg/dL)
**PBS**	6	114.8 ± 6.00	78.10 ± 2.77	11.32 ± 1.09	242.0 ± 18.62	166.0 ± 8.52
**NC26**	7	116.1 ± 3.49	80.73 ± 2.34	11.29 ± 0.57	269.3 ± 13.89	159.7 ± 9.93
**C26-noncx**	7	105.1 ± 9.043	77.85 ± 2.75	10.38 ± 0.50	262.4 ± 18.99	164.1 ± 17.41
**P value** (statistical test used)		**0.3578** (Kruskal-Wallis)	**0.6872** (ANOVA)	**0.5740** (Kruskal-Wallis)	**0.5369** (ANOVA)	**0.9451** (ANOVA)
**PBS**	7	111.4 ± 4.15	94.59 ± 3.62	6.83 ± 0.40	242.0 ± 17.73	65.00 ± 5.05
**NC26**	9	116.1 ± 2.68	93.72 ± 2.08	11.89 ± 0.65 **	212.1 ± 12.95	57.78 ± 3.62
**C26-cx**	6	127.0 ± 3.16 *	96.60 ± 2.90	19.85 ± 1.63 **** §§§§	164.2 ± 8.16 **	61.67 ± 10.44
**P value** (statistical test used)		**0.0175** (ANOVA)	**0.7758** (ANOVA)	**<0.0001** (ANOVA)	**0.0056** (ANOVA)	**0.6934** (ANOVA)
**PBS**	10	118.6 ± 4.21	85.66 ± 3.54	9.50 ± 0.29	230.1 ± 19.20	69.40 ± 6.49
**C26-precx**	11	120.0 ± 3.96	80.74 ± 1.92	17.03 ± 1.55 *	209.2 ± 18.62	71.45 ± 6.15
**C26-cx**	9	163.1 ± 9.62 **** §§§§	99.83 ± 4.70 * §§	34.90 ± 3.22 **** §§§§	165.7 ± 18.49	65.33 ± 5.14
**P value** (statistical test used)		**<0.0001** (ANOVA)	**0.0017** (ANOVA)	**<0.0001** (ANOVA)	**0.0749** (ANOVA)	**0.7738** (ANOVA)
**PBS**	5	79.60 ± 4.47	56.13 ± 2.62	13.06 ± 0.57	311.6 ± 6.82	113.8 ± 8.97
**LLC**	6	88.67 ± 4.85	57.42 ± 3.20	22.00 ± 0.82 ****	322.3 ± 21.68	83.83 ± 5.80 *
**P value** (statistical test used)		**0.2095** (*t* test)	**0.7702** (*t* test)	**<0.0001** (*t* test)	**0.6746** (*t* test)	**0.0175** (*t* test)
**WT**	9	106,9 ± 5,24		11,24 ± 0,46	385,0 ± 27,99	67,67 ± 4,84
**APC$^{Min/+}$**	8	205,9 ± 18,59 ****		55,40 ± 3,66 ****	217,5 ± 16,65 ***	867,3 ± 130,3 ****
**P value** (statistical test used)		**<0.0001** (*t* test)		**<0.0001** (*t* test)	**0.0002** (*t* test)	**<0.0001** (*t* test)
